# Development and validation of medication assessment tools to evaluate prescribing adherence to evidence-based guidelines for secondary prevention of coronary heart disease in post-acute coronary syndromes patients in Kuwait

**DOI:** 10.1371/journal.pone.0241633

**Published:** 2020-11-30

**Authors:** Dalal Al-Taweel, Abdelmoneim Awad

**Affiliations:** Department of Pharmacy Practice, Faculty of Pharmacy, Kuwait University, Jabriya, Kuwait; Fondazione Toscana Gabriele Monasterio & Scuola Superiore Sant'Anna, Pisa, Italy, ITALY

## Abstract

Cardiovascular diseases are estimated to cause 46% of all mortalities in Kuwait. The aim of evidence-based clinical practice has led to an increased interest in the design of medication assessment tools (MATs) to identify deviations from evidence-based practice, and eventually provide the basis of consistent standardized prescribing. This study was designed to develop and validate MATs using quality standards extracted from international guidelines to evaluate prescribing practices in secondary prevention of coronary heart disease in patients with post-acute coronary syndrome (STEMI or NSTEACS]. International guidelines were reviewed to develop two MATs (MAT_STEMI_ and MAT_NSTEACS_). Face and content validity of the developed tools was performed with three MAT experts and thirteen cardiologists. Two quantitative approaches were used to determine content validity: (i) Content Validity Ratio (CVR) and the average of CVR values; and (ii) Content validity index at item level (I-CVI) and scale-level of the tool (S-CVI/Ave) with the average approach. Criteria with a CVR<0.54 and I-CVI <70% were eliminated. Ultimately, feasibility testing of both MATs was performed on 66 patients’ records as a pilot study. The initial developed MAT_STEMI_ and MAT_NSTEACS_ consisted of eighteen and twelve medication-related criteria, respectively. Face validity resulted in dividing each MAT into five dimensions. In the MAT_STEMI_, three criteria had CVR values < 0.54 and I-CVIs < 70%. Two criteria were eliminated and one was retained. This resulted in sixteen criteria with average CVR 0.85 and S-CVI/Ave 92.3%. In the MAT_NSTEACS_, one criterion was eliminated. This resulted in eleven criteria with average CVR 0.93 and S-CVI/Ave 96.5%. The overall adherence scores to the MAT_STEMI_ and MAT_NSTEACS_ were 64.1% (95% CI: 57.8–69.9%) and 62.0% (95% CI: 53.4–69.9%), respectively. It was judged as intermediate adherence for both MATs. MAT_STEMI_ and MAT_NSTEACS_ were developed and validated to be utilized for optimizing medication therapy management and improving therapeutic interventions.

## Introduction

The definition of clinical guidelines has been stated as “*systematically developed statements to assist practitioner and patient decisions about appropriate health care for specific clinical circumstances*” [1]. They are valuable in reducing the risks of providing unequal care to patients as they allow practitioners to provide systematic and efficient care to their patients, as well as keeping their decisions in line with published evidence-based clinical outcomes [2]. Evidence suggests that the use of standardized guidelines to guide and implement best practice is associated with improvements in the efficacy and safety of medical care, as well as cost-effectiveness [3, 4]. Moreover, studies have shown positive effects of adherence to guidelines on patients’ health care outcomes [5, 6].

Quantification of guideline adherence serves as an outcome measure to evaluate the influence of services on quality of medication use. It offers a new approach to the assessment of prescribing practices and could serve as an example for many disease states. Medication assessment tools (MATs) have been used as an audit tool to assess the therapeutic management of many diseases globally including cancer [7, 8], heart failure [9], and diabetes [10, 11]. MATs are developed using clear, defined standards endorsed by clinical guidelines and are designed to measure prescribing practices of clinicians to defined clinical guidelines by applying them to patients' medical records. They have the advantage of quantifying adherence to guidelines and ensuring more equal care at a low cost of application and a higher degree of fairness [12]. However, in this era of evidence-based medicine, those tools must be reviewed regularly when guidelines are updated, to reflect the most recent evidence and up-to-date recommendations. In the Gulf Cooperation Council (GCC) region, healthcare managers have expressed an increased interest in the improvement of the quality of prescribing, and this can be implemented by the development of tools that identify and quantify deviations in prescribing practices to specific patient groups by using explicit standards extracted from published evidence-based clinical guidelines [11, 13, 14].

Cardiovascular diseases (CVDs) have been proven to be the number one cause of death worldwide and include coronary heart disease (CHD), cerebrovascular disease, peripheral arterial disease, rheumatic heart disease, congenital heart disease, deep vein thrombosis and pulmonary embolism [15]. CVDs in Kuwait cause approximately 46% of all mortalities [16, 17]. The effective prevention and management of chronic CVDs is a global priority and there is a need for change in the management of CVD and the regulation of care by healthcare professionals to offer reliable, best quality care consistently [18]. Translating the evidence behind CVD management into practice often proves to be difficult in the real world, as prescribers find themselves juggling between benefits/risks, published evidence, patients’ health needs and preferences when treating patients with multiple disease states [19]. Moreover, the pharmacotherapy management of different disease states to achieve desired therapeutic targets, such as for hypertension, hypercholesterolemia, and diabetes mellitus, is complex and time-consuming [20, 21]. This partially explains the existence of quality gaps in the management of patients with CVD worldwide [22, 23].

Evidence-based medication therapy and therapeutic goals for secondary prevention of CHD are well described and easily accessible in clinical practice guidelines [24–27]. However suboptimal prescribing is still a problem, and there is a substantial potential to foster the standard of preventive care to decrease the risk of recurrent cardiovascular events and death [28]. A potential method for detecting opportunities to enhance medication therapy management in secondary prevention of CHD is the extraction of medication quality standards from clinical guidelines, and hence the development of MATs, to identify and prevent deviations from evidence-based clinical practice. Previous studies reported that adhering to these quality standards results in improving patient outcomes and reducing all-cause mortality rates [29, 30]. Few studies have been performed in the developed countries using the MAT methodology to audit the secondary prevention of CHD, while there are no similar published studies from the developing countries [10, 31, 32]. There has been an increased interest in Kuwait in recent years in providing evidence-based clinical services by practitioners. In the Middle East and North Africa (MENA) region, including Kuwait, there is a lack of literature in prescribing practices of physicians for the treatment of CVDs. This information is necessary as a foundation for the development of a quality assurance framework aimed at ensuring optimal patient care. This study aimed to design and validate MATs using medication quality standards extracted from international clinical guideline to evaluate prescribing practices regarding secondary prevention of CHD in post-ACS patients (ST-Elevation Myocardial Infarction [STEMI] or non-ST elevation acute coronary syndrome [NSTEACS], which includes non-STEMI and unstable angina) in the outpatient clinics of healthcare facilities in Kuwait.

## Materials and methods

The development and validation of the MATs involved three steps: development of standards, face and content validation and feasibility testing.

### Development of standards

Due to the heterogeneous nature of physicians in Kuwait, a variety of guidelines are used to treat patients with cardiovascular diseases. Therefore, some exploratory work was done prior to the initiation of the study to determine what guidelines were most commonly used by physicians for secondary prevention of CHD in post-STEMI and post-NSTEACS patients. Three consultant cardiologists were contacted individually for an unstructured meeting, all of whom are heads of departments in their respective hospitals, and it was agreed that both, the European Society of Cardiology (ESC) and the American College of Cardiology/ American Heart Association (ACC/AHA) guidelines are the most followed guidelines in Kuwait. As the recommendations in both guidelines are divided into post-STEMI and post-NSTEACS, it was decided to develop two guideline-based MATs (MAT_STEMI_ and MAT_NSTEACS_) for this study. Medication-related criteria were chosen following an extensive literature review and an iterative process to identify relevant criteria from guideline recommendations of class I or IIa and the level of evidence A or B from the ESC guidelines [24, 25] and the ACC/AHA guidelines [26, 27]. Each criterion in the MAT is composed of two segments: qualifying statement and audit standard. The qualifying statement would initially be viewed in order to identify those patients eligible for the application of the standard. Application of the standard on eligible patients involves choosing an answer category from five different possible answers: Yes—the standard is met; no justified [No (J)]—the standard is not adhered to but an explicitly justified reason is present and documented in the patient’s notes; no unjustified [No (U)]—the standard is not adhered to and there is no explicitly apparent or documented reason in the patient’s notes; insufficient data qualifier [IDQ]—insufficient data on part of the qualifier; insufficient data standard [IDS]—insufficient data on part of the standard. If a patient is not eligible for the application of the standard, not applicable (NA} is recorded. These categorizations were used as both MATs will be implemented to calculate percentage adherence and percentage non-adherence to clinical guidelines.

The MAT_STEMI_ consisted of 18 medication-related criteria and the MAT_NSTEACS_ consisted of 12 medication-related criteria. The criteria of both MATs were designed in a Likert scale questionnaire containing five points to undergo face and content validity. Each criterion had five options (strongly agree, agree, disagree, strongly disagree, and not applicable).

### Face and content validity

Face validity was demonstrated through consultation with three MAT experts: an Assistant Professor of Pharmacy Practice at Qatar University (specializing in MAT development), a Professor of Pharmacy Practice at Kuwait University (specializing in cardiovascular diseases) and a Clinical lecturer at the University of Dundee (with extensive experience in MAT development in cardiovascular diseases).

Content validity was established through an electronic survey to an expert group comprising of 13 cardiologists working in Kuwait who were asked to indicate their level of agreement to each criterion on a 5-point Likert scale (strongly agree/agree/disagree/strongly disagree/not applicable). They were given three weeks to complete the survey. Two reminders (each one week apart) were also sent. The following two quantitative approaches were used to determine the content validity: (i) Content Validity Ratio (CVR) and the content validity index (average of the CVR values of all criteria) [33]; and (ii) Content validity index at the item level (I-CVI) and the scale-level of the assessment tool (S-CVI) with the average approach [3, 34, 35]. In the first approach, CVR for each criterion was calculated using the formula [CVR = (N_e_ - N/2)/(N/2), in which the N_e_ is the number of experts who agreed/strongly agreed on the respective item and N is the total number of the experts involved in the study. Based on Lawshe table, with the total number of 13 cardiologists in this study, the minimum CVR critical value for each criterion is 0.54 at α = 0.05. So, if the criterion obtained less than the critical value, it needs to be modified or deleted. The CVI was computed as the sum of the CVR values of all criteria, divided by the total number of criteria [33]. The second approach was the most widely reported for content validity in the development of instruments [3, 34, 36]. The content validity index at the item level (I-CVI) was calculated as the number of experts who agreed/strongly agreed to the relevancy of each criterion, divided by the total number of experts involved in the study. The I-CVI expresses the proportion of agreement on the relevancy of each criterion. The content validity index at the scale-level was determined using the average approach(S-CVI/Ave), in which the sum of I-CVIs is divided by the total number of criteria [34, 35, 37]. The recommended judgment on each item is as follows: If the I-CVI > 79%, the item is appropriate. If it is between 70% and 79%, it requires revision. If it < 70%, it should be deleted [38].

### Feasibility testing

Following face and content validation, the MATs underwent a feasibility assessment to ensure that they are fit for assessing prescribing adherence to evidence-based guidelines. The feasibility phase was a descriptive, cross-sectional study that was conducted on 66 patients’ medical records of patients attending the cardiovascular outpatient clinics in a secondary healthcare setting in Kuwait. As this was a feasibility study to pilot the developed MATs, the sample size calculation was not performed. Ethical approval was granted by the Standing Committee for Coordination of Health and Medical Research, Ministry of Health, Kuwait (Ethics No: 2018/866). Informed consent was not required as data was extracted from the patient’s medical records retrospectively. No patient identifiable information was collected to ensure patient confidentiality.

Patients included in this study were those who had experienced either STEMI or NSTEACS within at least 12 months preceding the study period, aged between 18 and 75 years, and had attended the cardiovascular outpatient clinic at the hospital, for the long-term management of STEMI or NSTEACS. Patients aged < 18 years or > 75 years were excluded because more conservative goals are often used for these patient groups. Also, pregnant or breastfeeding women and patients newly diagnosed with prior STEMI or NSTEACS, less than 12 months were excluded.

### Statistical analysis

Data were analyzed using the Statistical Package for Social Sciences (IBM SPSS Statistics for Windows, version 25, Armonk, NY: IBM Corp). The results were presented as percentages (95% confidence intervals; CI), means (standard deviation-SD) if data were normally distributed, and medians (Interquartile range-IQR) if data were not normally distributed. The content validity measures were computed as being described above. Pearson correlation was also used to analyze the association between the results of the two methods used to determine content validity. Percentage adherence to every single criterion as well as the overall percentage adherence for each MAT was calculated from the summation of the total number of cases where the standard is adhered to (Yes answers) over the summation of the total number of applicable cases, where the standard should be adhered to (Yes, No(U) and IDS answers) [(Σ Yes / Applicability) x 100]. Also percentages of unjustified non-adherence and justified adherence were calculated as follows: (i) percentage unjustified non-adherence = [No(U) / Applicability] x 100 and (ii) percentage Justified non-adherence = [No(J)] / [Applicability + No(J)] x 100. The criteria adherence was judged using arbitrary cut-offs based on a previous study of similar design (high, ≥ 80%; intermediate, 50 to 79%; low < 50%) [11].

## Results

### Development and validation of the Medication Assessment Tools (MATs)

Face validity of the initial MAT_STEMI_ and MAT_NSTEACS_ resulted in rewording, expanding and redefining some of the criteria, and dividing both MATs into five sections: i) antithrombotic therapy, ii) beta-blockers, iii) lipid-lowering therapy, iv) inhibitors of renin-angiotensin-aldosterone system and v) miscellaneous. This resulted in the first drafts of MAT_STEMI_ with a total number of 18 criteria and MAT_NSTEACS_ with a total number of 12 criteria.

The first drafts underwent content validation through an online survey to an expert group comprising of 13 cardiologists working in Kuwait, to generate the final draft of MAT_STEMI_ and MAT_NSTEACS_. Table 1 shows the characteristics of the expert group. The cardiologists’ level of agreement to the MATs criteria was evaluated using a 5-point Likert scale. Each statement was scored 4 for strongly agree, 3 for agree, 2 for disagree, and 1 for strongly disagree. Any statement that was not applicable was scored zero. The median (IQR), CVR, average of CVR values, I-CVI, and S-CVI/Ave were calculated and displayed in Tables 2 and 3. Any criterion that obtained a CVR < 0.54 and an I-CVI < 70% was eliminated from the MAT. Table 2 presents the cardiologists' responses to the MAT_STEMI_. Three criteria (10, 15, and 18) scored CVR values < 0.54 and I-CVIs < 70%. Following discussion with the research group, it was agreed that criteria 10 and 18 were to be eliminated; however, as criteria 15 is classified as Class I (Level of Evidence A) in ESC and ACC/AHA guidelines, it was decided to be retained in the MAT. The final draft of the MAT_STEMI_ consisted of a total number of 16 criteria with an average of CVR values 0.85 and SCVI/Ave 92.3%. Table 3 presents the cardiologists' responses to the MAT_NSTEACS_. One criterion number 12 had a CVR < 0.54 and an I-CVI < 70%, which was eliminated. The final draft of the MAT_NSTEACS_ consisted of a total number of 11 criteria with an average of CVR values 0.93 and SCVI/Ave 96.5%. Pearson correlation analysis of the content validity results of both approaches shows a high positive correlation (p <0.001).

**Table 1:** Demographic characteristics of the expert group (cardiologists) (n = 13).

Characteristic	Frequency (%)
Age (Years)	
**21–30**	1 (7.7%)
**31–40**	4 (30.8%)
**41–50**	1 (7.7%)
**51–60**	7 (53.8%)
Gender	
**Male**	12 (92.3%)
**Female**	1 (7.7%)
Years of practice	
**1–10**	5 (38.5%)
**11–20**	5 (38.5%)
**21–30**	2 (15.4%)
**>30**	1 (7.7%)
Rank	
**Registrar**	2 (15.4%)
**Specialist**	6 (46.2%)
**Senior specialist**	2 (15.4%)
**Consultant**	3 (23.1%)
Area of practice	
**Ministry of health**	12 (92.3%)
**Academia + Ministry of Health**	1 (7.7%)

**Table 2:** Expert group responses to the MAT_STEMI_ (n = 13).

NO	Statement	Number of respondents (%)							
		*SA n (%)	A n (%)	D n (%)	SD n (%)	NA n (%)	Median (IQR)	CVR	I-CVI (%)
***Antithrombotic therapy***									
***1***	All patients are prescribed aspirin 81-325mg, indefinitely (if no contraindication).	11(84.6)	2(15.4)	-	-	**-**	4 (4,4)	1	100
***2***	Patients with hypersensitivity to aspirin, are prescribed clopidogrel 75mg	11(84.6)	2(15.4)	-	-	**-**	4 (4,4)	1	100
***3***	For patients with stents post primary PCI, in addition to aspirin 81mg (if not contraindicated), are prescribed clopidogrel 75mg or ticagrelor 90mg BID daily for at least 12 months–dual therapy	13(100)	-	-	-	**-**	4 (4,4)	1	100
***4***	For patients post fibrinolysis, WITHOUT subsequent PCI, are prescribed clopidogrel 75mg daily in addition to aspirin for at least 14 days and up to 1 year in absence of bleeding.	10(76.9)	2(15.4)	1(7.7)	-	**-**	4 (4,4)	0.85	92.3
***5***	Patients post fibrinolysis WITH subsequent PCI, are prescribed clopidogrel 75mg daily in addition to aspirin for 12 months	11(84.6)	1 (7.7)	1(7.7)	-	**-**	4 (4,4)	0.85	92.3
***6***	A proton pump inhibitor should be prescribed for patients on dual antiplatelet therapy and at higher than average risk of gastrointestinal bleeding.	11(84.6)	2(15.4)	-	-	**-**	4 (4,4)	1	100
***Beta-Blockers***									
***7***	All post STEMI patients (with no contraindications to β-blockers) are prescribed β-blockers	8(61.5)	4(30.8)	1(7.7)	-	**-**	4 (3,4)	0.85	92.3
***8***	Patients with no contraindications to β-blockers and prescribed a β-blocker; are prescribed either metoprolol succinate SR, bisoprolol, or carvedilol for up to 3 years	8(61.5)	4(30.8)	1(7.7)	-	**-**	4 (3,4)	0.85	92.3
***9***	Patient with no contraindications to beta blockers with LVEF ≤ 40% are prescribed a beta-blocker either metoprolol succinate SR, bisoprolol, orrr carvedilol, indefinitely.	8(61.5)	4(30.8)	1(7.7)	-	**-**	4 (3,4)	0.85	92.3
***10***	Patients with contraindication to β-blockers (without LVEF≤ 40%), are prescribed non-dihydropyridine calcium channel blockers: verapamil or diltiazem.	3(23.1)	4(30.8)	5(38.5)	1(7.7)	**-**	3 (2,3)	0.08	53.8
***Lipid-lowering therapy***									
***11***	All patients (with no contraindication to statins) regardless of lipid levels are prescribed a high intensity statin (atorvastatin 40-80mg, rosuvastatin 20-40mg).	9(69.2)	4(30.8)	-	-	-	4 (3,4)	1	100
***12***	Atorvastatin 80mg is the drug of choice for patients.	4(30.8)	7(53.8)	1(7.7)	1(7.7)	-	3 (3,4)	0.69	84.6
***13***	Patients on statins should have LDL cholesterol < 1.8 mmol/L /at least 50% reduction	10(76.9)	2(15.4)	-	-	1(7.7)	4 (4,4)	0.85	92.3
***14***	Patients with LDL ≥ 1.8 mmol/L and despite a maximally tolerated statin, should be on further therapy (ezetimibe).	9(69.2)	3(23.1)	-	-	1(7.7)	4 (3,4)	0.85	92.3
***Inhibitors of renin-angiotensin aldosterone system***									
***15***	All patients (with no contraindication to ACE inhibitors) are prescribed ACE inhibitors.	4(30.8)	4(30.8)	4(30.8)	-	1(7.7)	3 (2,4)	0.23	61.5
***16***	Patients with an intolerance to ACE inhibitors are prescribed ARBs.	6(46.2)	6(46.2)	1(7.7)	-	-	3 (3,4)	0.85	92.3
***17***	Patients already receiving an ACEI and beta blocker, and have LVEF ≤ 40%, and either heart failure or diabetes (without significant renal dysfunction, or hyperkalemia) are prescribed aldosterone antagonist.	7(53.8)	5(38.5)	-	1(7.7)	-	4 (3,4)	0.85	92.3
***Miscellaneous***									
***18***	All patients ≥65 years or with increased risk, including smokers, immunocompromised patients and those with asthma should be prescribed a pneumococcal vaccine within the last 5 years	6(46.2)	3(23.1)	2(15.4)	2(15.4)	-	3(3,4)	0.38	69.2
								**CVI	^+^S-CVI/Ave
								0.78	88.9%

*SA: strongly agree, A: agree, D: disagree, SD: strongly disagree, NA; not applicable

** Content validity index (average of the CVR values of the statements)

^+^ Content validity index at the scale-level using the average approach

**Table 3:** Expert group responses to the MAT_NSTEACS_ (n = 13).

NO.	Statement	Number of respondents (%)							
		*SA n(%)	A n(%)	D n(%)	SD n(%)	N/A n(%)	Median (IQR)	CVR	I-CVI (%)
***Antithrombotic therapy***									
1	All patients are prescribed aspirin 81-325mg, indefinitely (If no contraindication).	8(61.5)	4(30.8)	-	1(7.7)	-	4 (3,4)	0.85	92.3
2	Patients with hypersensitivity to aspirin are prescribed clopidogrel 75mg or ticagrelor 90mg BID.	8(61.5)	4(30.8)	1(7.7)	-	-	4 (3,4)	0.85	92.3
3	For patients treated with ischemic guided strategy, in addition to aspirin 81mg (if not contraindicated), are prescribed clopidogrel 75mg OD or ticagrelor 90mg BID for a duration of up to 12 months.	9(69.2)	3(23.1)	-	1(7.7)	-	4 (3,4)	0.85	92.3
4	For patients with stents, in addition to aspirin 81mg (if not contraindicated), are prescribed ticagrelor 90mg BID or clopidogrel 75mg daily for at least 12 months–dual therapy.	10(76.9)	2(15.4)	-	-	1(7.7)	4 (4,4)	0.85	92.3
5	Patients on dual antiplatelet therapy and at higher than average risk of gastrointestinal bleeding are prescribed a proton pump inhibitor.	10(76.9)	2(15.4)	1(7.7)	-	-	4 (4,4)	0.85	92.3
***Beta-Blockers***									
6	Patients with LVEF ≤ 40% are prescribed beta blockers (metoprolol succinate SR, bisoprolol, carvedilol)—(If no contraindications).	7(53.8)	6(46.2)	-	-	-	4 (3,4)	1	100
***Lipid-lowering therapy***									
7	All patients regardless of lipid levels are prescribed a high intensity statin (atorvastatin 40-80mg, rosuvastatin 20-40mg)—(If no contraindication).	11(84.6)	2(15.4)	-	-	-	4 (4,4)	1	100
8	All patients with LDL cholesterol ≥ 1.8 mmol/L despite a maximally tolerated statin dose, are on ezetimibe.	10(76.9)	3(23.1)	-	-	-	4 (4,4)	1	100
***Inhibitors of renin-angiotensin aldosterone system***									
9	All patients with confirmed LVEF ≤ 40% or heart failure, or hypertension or diabetes are prescribed an ACE inhibitor.	12(92.3)	1(7.7)	-	-	-	4 (4,4)	1	100
10	All patients with intolerance to ACE inhibitors with confirmed LVEF ≤ 40% or heart failure, or hypertension or diabetes., are prescribed ARBs.	11(84.6)	2(15.4)	-	-	-	4 (4,4)	1	100
11	All patients already receiving an ACEI and beta blockers, and have LVEF ≤ 40%, and either HF or diabetes (without significant renal dysfunction, or hyperkaliemia) are prescribed aldosterone antagonist.	8(61.5)	5(38.5)	-	-	-	4 (3,4)	1	100
***Miscellaneous***									
12	All patients’ ≥65 years of age or with increased risk, including smokers, immunocompromised patients and those with asthma should be prescribed a pneumococcal vaccine within the last 5 years.	8(61.5)	1(7.7)	3(23.1)	-	1(7.7)	4 (2,4)	0.38	69.2
								**CVI	^+^S-CVI/Ave
								0.89	92.4%

*SA: strongly agree, A: agree, D: disagree, SD: strongly disagree, NA; not applicable.

** Content validity index (average of the CVR values of the statements).

^+^ Content validity index at the scale-level using the average approach.

### Feasibility testing

Table 4 shows the characteristics of patients included in the feasibility study. Their mean (SD) age was 56.8 (9.9) years. Of the 66 patients, 84.8% were females and 81.8% were non-Kuwaitis.

**Table 4:** Patients’ demographic and other characteristics (n = 66).

Characteristic	Frequency (%)
Age (Years)	
**30–39**	3 (4.6)
**40–49**	13 (19.7)
**50–59**	21 (31.8)
**60–64**	16 (24.2)
**≥ 65**	13 (19.7)
Gender*	
**Male**	56 (84.8)
**Female**	9 (13.6)
Nationality	
**Kuwait**	12 (18.2)
**Non-Kuwaiti**	54 (81.8)
Smoking Status*	
**Yes**	20 (30.3)
**No**	18 (27.3)
Alcohol Use*	
**Yes**	0 (0.0)
**No**	38 (57.6)

* Percentage may not total a 100% due to some missing data.

Table 5 presents the clinical characteristics of the patients. Thirty-six patients (54.4%) were diagnosed with prior STEMI. Of these post-STEMI patients, 9 (25.0%; 95% CI: 12.7–42.5) were treated with fibrinolysis without subsequent PCI and stent, 15 (41.7%; 95% CI: 26.0–59.1) were treated with fibrinolysis with subsequent PCI and stent, and 12 (33.3%; 95% CI: 19.1–51.1) were treated with a PCI and stent. On the other hand, of the 30 post-NSTEACS patients, 11 (36.7%; 95% CI: 20.5–56.1) were treated with ischemic guided strategy only and 19 (63.3%; 95% CI: 43.9–79.5) were treated with PCI and stent. Nine (13.6%) patients had left ventricular ejection fraction of ≤ 40%. Of the 41 patients with available recent BP values, 22 (53.7%) of them had BP ≥ 130/ 80 mmHg. Of the 51 patients with recent cholesterol values, 41 (80.4%) and 38 (74.5%) of them had LDL-C ≥ 1.81 mmol/L and non- HDL-C ≥ 2.59 mmol/L, respectively.

**Table 5:** Clinical characteristics of patients (n = 66).

Characteristic	Frequency (%)
**Post-STEMI patients n (%)**	36 (54.5)
**Post-NSTEACS patients n (%)**	30 (45.5)
**Duration of diagnosis (Years) Mean (SD)**	4.3 (3.3)
Left Ventricular Ejection Fraction (%)*	
**≤ 40 n (%)**	9 (13.6)
**41–49 n (%)**	12 (18.2)
**≥ 50 n (%)**	43 (65.2)
BP (mmHg)*	
**SBP and DBP < 130/80 n (%)**	19 (29.8)
**SBP ≥130 and/or DBP ≥80 n(%)**	10 (15.2)
**SBP ≥140 and/or DBP ≥90 n (%)**	12 (18.2)
**Mean (SD) SBP/DBP**	134 (21) / 75 (13)
LDL-C (mmol/L)*	
**< 1.81 n (%)**	10 (15.2)
**≥ 1.81 n (%)**	41 (62.1)
**Mean (SD)**	2.56 (1.09)
Non-HDL-C (mmol/L)*	
**< 2.59 mmol/L n (%)**	13 (19.7)
**≥ 2.59 mmol/L n (%)**	38 (57.6)
**Mean (SD)**	3.33 (1.04)
Triglycerides (mmol/L)*	
**< 1.7**	28 (42.4)
**≥ 1.7**	13 (19.7)
**Mean (SD)**	1.78 (0.87)

* Percentage may not total a 100% due to some missing data.

Table 6 shows adherence scores to the MAT_STEMI_ criteria. The level of overall adherence was judged as intermediate adherence (64.1%; 95% CI: 57.8–69.9%). The percentage of overall unjustified non-adherence was 31.6% (95% CI: 26.137.8), while that of justified non-adherence was 5.2% (95% CI: 4.2–6.3). There was very low applicability (less than 10 patients) in criteria 4, 6, 9, 11, 15, 16; and criterion 2 was not applicable to any patients. These seven criteria were not included in the calculation of the overall adherence and non-adherence. Following the application of MAT_STEMI_ on 36 patients’ medication records, out of the 576 criteria, only 317 relevant criteria were investigated among the patients, of which 283 (89.3%; 95% CI: 85.2–92.4) were found to be applicable. There were 36 cases of insufficient data (ID), of which 24 (66.7%; 95% CI: 49.0–80.9%) were considered as IDQ and 12 (33.3%; 95% CI: 19.1–51.1%) were IDS.

**Table 6:** Adherence to the audit tool criteria for post-STEMI patients (n = 36).

No.	Criteria	Applicability	% Adherence (95% CI)
***Antithrombotic therapy***			
1	Patient with no contraindication to aspirin is prescribed a daily dose of aspirin 81-325mg, indefinitely	36	100 (88.0–99.8)
2	Patient who is not prescribed aspirin due to hypersensitivity is prescribed clopidogrel 75mg daily	0	-
3	Patient with stent post-primary PCI is prescribed clopidogrel 75mg or ticagrelor 90mg BID daily for at least 12 months, in addition to aspirin 81mg as a dual therapy	18	83.3 (57.7–95.6)
4	Patient post fibrinolysis, WITHOUT subsequent PCI is prescribed clopidogrel 75mg daily in addition to aspirin for at least 14 days and up to 1 year in absence of bleeding	7	57.1 (20.2–88.2)
5	Patient post fibrinolysis WITH subsequent PCI is prescribed clopidogrel 75mg daily in addition to aspirin for 12 months	13	92.3 (62.1–99.6)
6	Patient on dual antiplatelet therapy and at higher than average risk of gastrointestinal bleeding is prescribed a proton pump inhibitor	7	100 (56.1–98.7)
***Beta-Blockers***			
7	Patient with no contraindications to beta-blockers is prescribed beta-blocker	35	97.1 (83.4–99.9)
8	Patient with LVEF > 40% with no contraindications to beta-blockers and prescribed a beta-blocker is prescribed either metoprolol succinate SR, bisoprolol or carvedilol for up to 3 years	29	72.4 (52.5–86.6)
9	Patient with NO contraindications to beta-blockers with LVEF ≤ 40% and prescribed a beta-blocker is prescribed either a metoprolol succinate SR, bisoprolol, or carvedilol indefinitely	5	100 (46.3–98.1)
***Lipid-lowering therapies***			
10	Patient regardless of lipid level is prescribed a high-intensity statin either atorvastatin 40-80mg or rosuvastatin 20-40mg	36	16.7 (7.0–33.5)
11	Patient prescribed a high-intensity statin is prescribed atorvastatin 80mg	5	40.0 (7.3–83.0)
12	Patient maintained on statins with a baseline LDL level 1.8–3.5 mmol/L has achieved target LDL cholesterol < 1.8 mmol/L or at least 50% reduction in LDL cholesterol	35	22.9 (11.0–40.6)
13	Patient with LDL ≥ 1.8 mmol/L and despite a maximally tolerated statin should be on further therapy (ezetimibe)	20	
***Inhibitors of renin-angiotensin aldosterone system***			
14	Patient with no contraindication to ACE inhibitors is prescribed an ACE inhibitor	34	94.1 (78.9–99.0]
15	Patient not prescribed ACE inhibitor due to intolerance is prescribed ARB	1	100 (5.5–89.2)
16	Patient already receiving an ACEI and beta-blocker, and have LVEF ≤ 40%, and either heart failure or diabetes (without significant renal dysfunction, or hyperkalemia) is prescribed aldosterone antagonist	2	50.0 (2.7–97.3)

Table 7 presents the adherence to the MAT_NSTEACS_ criteria. The level of overall adherence was judged as intermediate adherence (62.0%; 95% CI: 53.4–69.9%). The percentage of overall unjustified non-adherence was 38.0% (95% CI: 30.1–46.6), while that of justified non-adherence was 0.7% (95% CI: 0.1–4.4). There was very low applicability (less than 10 patients) in criteria 6, 10, 11; and criterion 2 was not applicable to any patients. These four criteria were not included in the calculation of the overall adherence and non-adherence. Following the application of MAT_NSTEACS_ on 30 patients’ medication records, out of the 330 criteria, only 166 relevant criteria were investigated among the patients, of which 156 (94.0%; 95% CI: 88.9–96.9) were found to be applicable. There were 11 cases of insufficient data (ID), of which 9 (81.8%; 95% CI: 47.8–96.8%) were considered as IDQ and 2 (18.2%; 95% CI: 3.2–52.3%) were IDS.

**Table 7:** Adherence to the audit tool criteria for post-NSTEACS patients (n = 30).

No.	Criteria	Applicability	% Adherence (95% CI)
***Antithrombotic therapy***			
1	Patient with no contraindication to aspirin is prescribed a daily dose of aspirin 81-325mg, indefinitely	30	100 (85.9–99.7)
2	Patient who is not prescribed aspirin due to hypersensitivity is prescribed clopidogrel 75mg daily	0	-
3	Patient treated with ischemic guided strategy, in addition to aspirin 81mg (if not contraindicated) is prescribed clopidogrel 75mg OD or ticagrelor 90mg BID for a duration of up to 12 months	11	90.9 (57.1–99.5)
4	Patient with stent post primary PCI is prescribed clopidogrel 75mg or ticagrelor 90mg BID daily for at least 12 months, in addition to aspirin 81mg as a dual therapy.	18	88.9 (63.9–98.1)
5	Patient on dual antiplatelet therapy and at higher than average risk of gastrointestinal bleeding Is prescribed a proton pump inhibitor	10	60.0 (27.4–86.3]
***Beta-Blockers***			
6	Patient with LVEF ≤ 40% with no contraindications to beta-blockers and prescribed a beta-blocker is prescribed either metoprolol succinate SR, bisoprolol, or carvedilol	4	100 (39.6–97.7]
***Lipid-lowering therapies***			
7	Patient regardless of lipid levels is prescribed a high-intensity statin either atorvastatin 40-80mg or rosuvastatin 20-40mg	30	33.3 (17.9–52.9)
8	Patient with LDL ≥ 1.8 mmol/L and despite a maximally tolerated statin should be on further therapy (ezetimibe)	21	
***Inhibitors of renin-angiotensin-aldosterone system***			
9	Patient with confirmed LVEF ≤ 40% or heart failure, or hypertension or diabetes is prescribed an ACE inhibitor	22	72.7 (49.6–88.4)
10	Patient with intolerance to ACE inhibitors with confirmed LVEF ≤ 40% or heart failure, or hypertension or diabetes is prescribed ARB	7	57.1 (20.2–88.2)
11	Patient already receiving an ACEI and beta blocker, and have LVEF ≤ 40%, and either heart failure or diabetes (without significant renal dysfunction, or hyperkalemia) is prescribed aldosterone antagonist	3	66.7 (12.5–98.2)

## Discussion

To the best of our knowledge, this is the first study in the Middle East and North Africa (MENA) region to develop and validate MATs using medication standards extracted from international clinical guidelines to evaluate prescribing practices in secondary prevention of CHD in post-STEMI and post-NSTEACS patients. In the present study, MAT_STEMI_ and MAT_NSTEACS_ were developed and validated as standardized tools to document and identify non-adherence to guidelines for future initiatives for quality improvements in routine practice. Also, these MATs may serve as valuable research tools to identify areas for improvement and monitor changes in adherence to clinical guidelines in Kuwait.

The importance of validity in tool development is an essential step in the development of our MATs. Face validity ensures that the tools developed read well and appear pleasant for respondents, and thus more acceptable. Some researchers claim that face validity is not considered as validity as far as measurement principles are concerned, since it does not consider what to measure, but focuses on the appearance of the tools [39]. However, it is deemed as a necessary step in the development of the present tools as MATs are quite complex tools to develop. We believe that our choice of the experts for face validity, being MAT experts in different disease states, ensured that the items were well refined and organized in a suitable format and sequence to enable data collection in a usable form. Content validity is a critical step in the development of an instrument to address the degree to which items of an instrument sufficiently represent the content domain and should be obtained on each developed instrument [40]. Quantifying content validity ensures that the tool measures knowledge of the content domain of which it was designed to measure knowledge. Previous MAT studies often established content validity through qualitative expert reviews. In the present study, two quantitative approaches were used to ensure the validity of the developed tools. A distinction in the results of different approaches used in the analysis of the content validity of the same instrument was reported in the literature [41]. The current results revealed that the final drafts of MAT_STEMI_ and MAT_NSTEACS_ enjoy an appropriate level of overall content validity with high values of average CVR (0.85 and 0.93, respectively) and SCVI/Ave (92.3% and 96.5%, respectively). The present study showed the agreement of CVR and SCVI/Ave analysis on the items of both MATs and their results were found to be positively correlated (p<0.001). Hence, it can be stated that both approaches are in line for decision-making on valid and invalid items in this study. Heterogeneity and the number of experts are considered to be crucial in the determination of the content validity of an instrument to decrease the probability of chance agreement [42]. The present study comprised 13 cardiologists including registrars, specialists, senior specialists, and consultants from different hospitals to represent potential different therapy traditions, particularly in the absence of intrinsic locally generated guidelines. Furthermore, the e-mail communication minimized time consumption and maintained anonymity between the expert panel members that may enable members to front their opinion regardless of the other members’ judgments.

The feasibility testing was performed retrospectively on 66 patients’ medical records in the outpatient clinics as a pilot study to test the MATs fitness for purpose. The present findings of the pilot study revealed that both MATs performed very well in a clinical setting and were able to identify good clinical performances as well as improvement potentials regarding secondary prevention of CHD in post-ACS patients. Post-ACS patients, regardless of nationality, are followed up every two months in the outpatient cardiovascular clinics in secondary care in Kuwait. The overall criteria applicability of MAT_STEMI_ and MAT_NSTEACS_ were 89.3% and 94%, respectively, which are high compared to those indicated in other studies using the MAT methodology [7, 10, 43]. Criterion 2 in both MATs was not applicable in the study population. Also, six criteria in the MAT_STEMI_ and three criteria in the MAT_NSTEACS_ had low applicability (less than 10 patients). This could be attributed to the small sample size and convenience sampling from a single hospital in this pilot study. However, when utilizing the MATs prospectively as clinical tools, even low-applicability criteria are important because their exclusion may increase the risk of overlooking crucial aspects of management in a few eligible patients. Also, they can serve as reminders of guideline recommendations in rare clinical situations. Therefore, inclusion and exclusion of criteria should not exclusively concern applicability but also the clinical relevance [32]. The total adherence scores to the MAT_STEMI_ and MAT_NSTEACS_ applicable criteria were judged as intermediate for both MATs, which is similar to that reported in studies from developed countries using the MAT methodology to audit the secondary prevention of CHD [10, 31, 32]. Several factors could contribute to the intermediate adherence score achieved in this study, including, the lack of national guidelines for the management of chronic cardiovascular disorders, as well as the limited multisiciplinary approach to the management of patients with chronic cardiovascular disorders in Kuwait which makes it more difficult to adapt clinical standards into practice [44]. However, as this was a feasibility study, a larger study with a representative sample is needed to confirm the adherence score. Insufficient documentation (recorded as IDQ and IDS) was apparent for both MATs, which is consistent with previous MAT studies conducted in Kuwait to evaluate prescribers’ adherence to evidence-based guidelines in type 2 diabetes mellitus and bipolar disorder [11, 14] that highlighted a clear gap in documentation in medical records, particularly in the recording of laboratory results. This could lead to a possible low estimate of adherence and does not necessarily indicate a lack of care. This highlights the need for adequate documentation to ensure optimum continuity of care between healthcare settings and personnel. Also, the MAT_STEMI_ and MAT_NSTEACS_ may be valuable in revealing gaps in documentation practice in patients’ records at healthcare facilities.

The present findings revealed low achievement of optimal target goals for BP, LDL-C, and non-HDL-C among the study population. This is alarming and increases the risk for recurrent cardiovascular events. Factors reported as limitations to patients achieving target goals included inappropriate prescribing practices, poor patient adherence to medication, and unawareness of the importance of therapeutic goal attainment [45].

### Strengths and limitations

The developed and validated MATs were found to be valid with respect to feasibility in a Kuwaiti hospital setting. These novel MATs would be beneficial in routine practice to facilitate appropriate prescribing by helping clinicians discuss local practice by reference to evidence-based guidelines and also help identify areas that should be proposed to quality improvement initiatives. In addition, the MATs developed will be applied as research tools in the future in a large intervention study as outcome measures to assess the adherence of prescribers to evidence-based guidelines for secondary prevention of CHD in the outpatient clinics of healthcare facilities in Kuwait.

The present study has certain limitations. MAT_STEMI_ and MAT_NSTEACS_ were developed using the European and American guidelines, the ESC guidelines [24, 25], and the ACC/AHA guidelines [26, 27] that are in accordance with the guidelines used by the cardiologists in the secondary prevention of CHD in post-ACS patients in Kuwait. Although both MATs were developed for a Kuwaiti setting, they can be applied in different countries and settings, with adaptations to local and national guidelines, in addition to clinical cultures [46]. The small sample size and convenience sampling from a single hospital, which are regarded as sufficient for the main aim of the feasibility study to test the MATs fitness for purpose, would have affected the external validity in terms of generalizing the findings to the wider population. Even though standardized predefined data collection forms were used to extract data from patients’ records, the application time was not measured to inform the clinical utility of the MATs in a real world setting. However, the time used can be comparable with that utilized in data collection for medication review in practice settings.

### Conclusions

A 16-criterion MAT_STEMI_ and an 11-criterion MAT_NSTEACS_ were developed from international clinical guidelines to help practitioners to standardize the medication review process in secondary prevention of CHD in post ACS patients. The criteria in both MATs are explicitly defined, face and content validity, and feasibility were explored with appropriate results indicating that both MATs include necessary aspects of quality of care of secondary prevention of CHD in post ACS patients. The feasibility study indicated that a retrospective chart review using both MATs on a larger scale will enable identification of good clinical performance as well as potential areas for improvement. Hence, these MATs will serve as tools for quality assurance of medication therapy in clinical settings and will help in the establishment of standards for prescribing practice. Those tools must be reviewed regularly when guidelines are updated, to reflect the most recent evidence and up-to-date recommendations.

## Supporting information

S1 Dataset (SAV)
